# 2021 Simulation Summit Webinar Series: Virtual. 17-18 February 2021

**DOI:** 10.1186/s41077-021-00159-z

**Published:** 2021-03-31

## 01 Creating psychological safety in interprofessional simulation: A scoping review

### Kelly Lackie^1^, Kathryn Hayward^1^, Caitlyn Ayn^1^, Peter Stilwell^2^, Cynthia Andrews^1^, Noel Pendergast^1^, David Persaud^1^, Shannan Grant^3^, Shauna Houk^1^, Marion Brown^1^, Jonathan Harris^1^, Sheri Price^1^, Doug Ferkol^1^, Jacquie Thillaye^1^, Jessica Mills^4^

#### **Correspondence:** Kelly Lackie

##### ^1^Dalhousie University, Halifax, NS, Canada; ^2^McGill University, Montreal, QC, Canada; ^3^Mount Saint Vincent University, Halifax, NS, Canada; ^4^IWK Health Centre, Halifax, NS, Canada

**Background**

Simulation-based education (SBE) is used to augment and/or partially substitute clinical learning by providing controlled settings with natural, realistic, and relevant practice experiences, without risk of injurious patient consequences. Interprofessional SBE (IP-SBE) offers a forum for students to learn about, from, and with one another to develop/practice interprofessional collaborative (IPC) competencies. However, socio-historical hierarchies that are predominant in healthcare often emerge in simulation, negatively impacting interprofessional learning. Creating conditions of psychological safety in IP-SBE can address the subtle coercive practices that exist amidst the power differentials within healthcare and facilitate fuller participation of students in their application of IPC competencies.

**Objective**

A scoping review was conducted to better understand the barriers and enablers to implementing psychologically safe IP-SBE by addressing the question: What are the barriers and enablers to creating a psychologically safe environment to effectively facilitate acquisition and application of the IPC competencies within IP-SBE?

**Materials and methods**

An interprofessional team of researchers and educators, representing nine health professions, as well as simulated patient educators (SPEs) conducted the scoping review. The scoping review included English language peer-reviewed empirical studies, grey literature, and theoretical articles published after Jan 1, 1990 that addressed psychological safety within uniprofessional and IP-SBE. Screening and full text review were completed based on inclusion/exclusion criteria, followed by data extraction. Each stage of the review was completed by two team members. Conflicts were resolved by the PIs.

**Results**

There were 1653 studies screened; 1527 were deemed irrelevant. After full text review, 98 more were excluded that did not discuss psychological safety and/or simulation. The remaining 27 studies were analyzed. Enablers of psychological safety included pre-briefing, creating a no-blame culture, and standardized debriefing by trained facilitators. Barriers included hierarchy, interprofessional stereotypes, and deliberate misdirection of simulations.

**Conclusions**

IP-SBE, facilitated in ways that are non-threatening, supportive, non-judgmental, while treating mistakes as learning opportunities create conditions for psychologically safe learning. Identifying barriers and enablers of psychologically safe IP-SBE is crucial for effective learning of IPC competencies, a necessity for the development of health care professionals who are able and willing to collaborate to provide safe, quality patient-centered care.

## 02 Variable meanings of entrustment – variable decision-making? How supervisors make procedural entrustment decisions in simulation- and workplace-based settings

### Thurarshen Jeyalingam^1^, Catharine M Walsh^2^, Walter Tavares^3^, Maria Mylopoulos^4^, Kathryn Hodwitz^5^, Louis WC Liu^4^, Ryan Brydges^5^

#### **Correspondence:** Thurarshen Jeyalingam

##### ^1^University of Calgary, Calgary, AB, Canada; ^2^Hospital for Sick Children, Toronto, ON, Canada; ^3^University of Toronto, Wilson Centre, Toronto, ON, Canada; ^4^University of Toronto, Toronto, ON, Canada; ^5^Allan Waters Family Simulation Centre, St. Michael’s Hospital, Unity Health Toronto, Toronto, ON, Canada

**Background**

Entrustment, a central construct in competency-based medical education (CBME), represents the point at which clinical supervisors trust a trainee to perform a task independently. Many implementations of CBME involve asking busy supervisors to assess residents’ entrustment through observing entrustable professional activities (EPAs). While EPAs are frequently assessed in both simulation- and workplace-based settings, research has yet to clarify how supervisors form judgments when assessing the same EPA in these two settings.

**Objective**

We aimed to explore the features supervisors report as influencing their entrustment decisions when observing endoscopic polypectomy performance in the simulation- and workplace-based assessment settings.

**Material and methods**

We designed an interview-based, constructivist grounded theory-informed study involving gastroenterology supervisors and trainees at the University of Toronto academic hospitals. Supervisors completed separate EPA assessments of each trainee’s endoscopic polypectomy performance (a relevant speciality-specific EPA) in both workplace- and simulation-based settings, which occurred in that sequence approximately 3-weeks apart. Supervisors were interviewed after each to explore how they made their entrustment decision within and across settings. Our team coded the transcribed interview data iteratively using constant comparison to generate themes.

**Results**

Based on 14 interviews with 7 supervisors, we found that participants: (1) held multiple meanings of entrustment, both within and across participants, (2) expressed variability in how they justified their entrustment decisions, the related narrative, and numerical EPA assessment scoring, (3) held unique personal criteria for making entrustment decisions ‘comfortably’ (authenticity of the task, task-related variability, and opportunity to assess trainee response to unexpected events such as procedural complications), and (4) perceived a relative freedom when using simulation to make entrustment decisions due to the absence of a real patient.

**Conclusions**

We found that participants spoke about and defined entrustment in a variety of ways. That variety appeared to lead to variability in how supervisors judged entrustment, both within and across participants and settings. The observed rater idiosyncrasies suggest residency programs cannot assume equivalence of EPA assessment data from simulation- and workplace-based settings. Furthermore, educators designing faculty development for CBME likely need to attend to the criteria that supervisors report needing to comfortably make entrustment decisions.

## 03 The McGill World Restart a Heart 2020 Campaign: a student-led, patient-engaged, large-scale online simulation program

### Jeremy Y Levett^1^, Simon Bichara-Allard^1^, Elodie Chamass^1^, Sirin Chami^1^, Alexa Ehlebracht^1^, Sarah Elbaz^1^, Amanda Essebag^1^, Christina Skiadopoulos^1^, Diana Colby^1^, Francois de Champlain^2^, Katrysha Gellis^3^, Mathew Hannouche^4^, Elene Khalil^4^, Diane Weidner^5^, Laura Winer^6^, Sandra Zambon^7^, Maggie Lattuca^8^, Karen Brown^9^, Nancy Posel^10^, Farhan Bhanji^11^

#### **Correspondence:** Jeremy Y Levett

##### ^1^Faculty of Medicine and Health Sciences, McGill University, Montreal, QC, Canada; ^2^McGill University Health Centre, Montreal, QC, Canada; ^3^Living Proof CPR Training, Montreal, QC, Canada; ^4^McGill University Health Centre, Montreal, QC, Canada; Steinberg Centre for Simulation and Interactive Learning, Montreal, QC, Canada; ^6^Teaching and Learning Services, McGill University, Montreal, QC, Canada; ^7^Heart and Stroke Foundation of Canada, Montreal, ON, Canada; ^8^Teaching and Learning Services, McGill University, Montreal, QC, Canada; ^9^Patient Education Office, McGill University Health Centre, Montreal, QC, Canada; ^10^McGill University, Montreal, QC, Canada; ^11^Montréal Children's Hospital, Montréal, QC, Canada

**Background**

Cardiac arrest is a leading cause of death in Canada, with the majority of cases occurring in homes or public places. Up to 55% are witnessed by family members, colleagues or friends, yet most victims don’t receive cardiopulmonary resuscitation (CPR), which can triple the odds of survival. Mortality therefore remains high at over 90%. Enhancing bystander CPR skills is a crucial public health investment.

**Objective**

Engage Canadians and the global community in a thought-provoking, immersive experience to explore cardiac arrests, destigmatize bystander CPR, and enhance resuscitation knowledge and skills. Partner with patients, leverage innovative technologies, and social media, to develop a digital scalable simulation/educational exercise.

**Materials and methods**

Interprofessional healthcare students from the Faculty of Medicine and Health Sciences of McGill University coordinated the McGill World Restart a Heart (WRAH) 2020 Campaign as part of a global initiative. Amplified by social media influencers, the Campaign team produced a bilingual digital Campaign with succinct and high-impact videos including survivor and non-survivor family perspectives, digital live events, a patient education guide for families of cardiac arrest patients, and an open access eModule. Our Steering Committee comprised University leadership, intensive and emergency care physicians, a cardiac arrest survivor, and the communications team from the Faculty, Steinberg Centre for Simulation and Interactive Learning, and McGill University Health Centre (MUHC).

**Results**

The Campaign garnered an international audience with over 74,636 impressions across social media platforms (Facebook, Instagram, and Twitter), 42,600 views of Campaign videos, and 1,500 views of hour-long webinars. Campaign ambassadors had a combined viewership of over 500,000 followers, and included astronaut physicians, Olympic athletes, adult and pediatric cardiac arrest survivors, and social media influencers. The Campaign is launching an innovative bystander resuscitation eModule that will deliver scalable screen-based simulation. The patient education guide, in collaboration with the MUHC’s Patient Education Office, focuses on the chain of survival and expectations for acute management, hospitalization and recovery. Visit the Campaign website (www.mcgill.ca/wrah) for more information.

**Conclusions**

Tens of thousands of individuals reached through the McGill WRAH 2020 Campaign are now empowered to initiate CPR, which may help reduce the morbidity and mortality of cardiac arrests.

## 04 Unpacking novice learners’ experiences with cognitive load during simulation-based training

### Faizal Haji^1^, Heather Braund^1^

#### **Correspondence:** Heather Braund

##### ^1^Queen's University, Kingston, ON, Canada

**Background**

Cognitive Load Theory (CLT) is a well-recognized framework used to guide instructional design within health professions education, particularly during simulation-based education. Despite the evident gains made in understanding the impact of cognitive load during the learning process, more research is required to clarify the extent to which instructional design features impact cognitive load and learning in simulated environments.

**Objective/Goal/Hypothesis/Research Question**: This qualitative study describes medical students’ experiences with cognitive load in a simulated learning environment. Specifically, we sought to identify sources of cognitive load, strategies for managing cognitive load, and the realism of the simulated environment as perceived by these novice learners.

**Materials and methods**

This study involved a secondary analysis of interview data that was collected as part of prior simulation research. Medical students (*n =* 109) from two Canadian institutions were recruited to participate in either ‘simple’ or ‘complex’ Lumbar Puncture training tasks. Students engaged in repetitive trials interspersed with expert feedback. We conducted post-training semi-structured interviews to understand participants’ experiences with the task and strategies used to manage their cognitive resources. Data were analyzed in NVivo using an emergent thematic approach.

**Results**

Five themes emerged from the data, the first three of which are detailed below (due to space limitations). The first theme identified the main sources of cognitive load, including purposefully embedded distractions (e.g. beeping, background noises), time constraints, and application of new knowledge without previous experience. The second theme described design features that facilitated learning, e.g. ability to communicate with the patient, receiving feedback, repetition, and preparation materials. The third theme highlighted learners’ load management strategies, including incorporating feedback, rehearsing next steps, staying calm, self-questioning or self-talk, prioritizing, ignoring distractions, and focusing on one skill or weakness at a time.

**Conclusions**

Findings suggested that participants were affected to varying degrees by purposefully integrated instructional design features. Students describe some key strategies used to manage their cognitive resources that have multiple implications for learning outcomes from training. Overall this study was able to better understand the facilitators and challenges to learning experienced by novices training in a simulation environment informed by Cognitive Load Theory.

